# Construction and Validation of a Digital Twin-Driven Virtual-Reality Fusion Control Platform for Industrial Robots

**DOI:** 10.3390/s25134153

**Published:** 2025-07-03

**Authors:** Wenxuan Chang, Wenlei Sun, Pinghui Chen, Huangshuai Xu

**Affiliations:** School of Intelligent Manufacturing Modern Industry, Xinjiang University, Urumqi 830046, China; cwxuan@stu.xju.edu.cn (W.C.); 107552304235@stu.xju.edu.cn (P.C.); 107552304352@stu.xju.edu.cn (H.X.)

**Keywords:** digital twin, Unity3D, industrial robotics, reality–virtual mapping, socket communication, human–computer interaction

## Abstract

Traditional industrial robot programming methods often pose high usage thresholds due to their inherent complexity and lack of standardization. Manufacturers typically employ proprietary programming languages or user interfaces, resulting in steep learning curves and limited interoperability. Moreover, conventional systems generally lack capabilities for remote control and real-time status monitoring. In this study, a novel approach is proposed by integrating digital twin technology with traditional robot control methodologies to establish a virtual–real mapping architecture. A high-precision and efficient digital twin-based control platform for industrial robots is developed using the Unity3D (2022.3.53f1c1) engine, offering enhanced visualization, interaction, and system adaptability. The high-precision twin environment is constructed from the three dimensions of the physical layer, digital layer, and information fusion layer. The system adopts the socket communication mechanism based on TCP/IP protocol to realize the real-time acquisition of robot state information and the synchronous issuance of control commands, and constructs the virtual–real bidirectional mapping mechanism. The Unity3D platform is integrated to develop a visual human–computer interaction interface, and the user-oriented graphical interface and modular command system effectively reduce the threshold of robot use. A spatially curved part welding experiment is carried out to verify the adaptability and control accuracy of the system in complex trajectory tracking and flexible welding tasks, and the experimental results show that the system has high accuracy as well as good interactivity and stability.

## 1. Introduction

Robots have emerged as representative devices embodying automation, intelligence, and digitalization, playing a pivotal role across various manufacturing sectors [1]. Compared to conventional technologies, robots demonstrate significant advantages in material handling, spraying, welding, cladding, and other applications—not only reducing reliance on manual labor but also substantially improving production efficiency [2,3,4,5,6]. In recent years, with the successive introduction of strategic initiatives such as Germany’s Industry 4.0 and Made in China 2025, the transition toward intelligent manufacturing has become a dominant global trend. Consequently, the application and advancement of robotic technologies have garnered widespread attention worldwide.

Although industrial robots have demonstrated remarkable success in manufacturing, they still face several limitations and developmental bottlenecks. Due to significant differences in control logic, programming languages, and communication protocols among robots from different brands, interoperability is poor, and standardization levels remain low, making it difficult for enterprises to achieve unified management and deployment. Moreover, traditional robot control methods suffer from limited programming visualization, making it challenging to accurately represent subtle variations during machining. This restricts control precision and robustness, while equipment failures can only be diagnosed on-site, resulting in high maintenance costs and slow response times. The integration of digital twin technology has the potential to significantly enhance the operational efficiency of industrial robots while simultaneously reducing the learning curve associated with their deployment and use [6,7,8,9]. By enabling deep integration between virtual and physical environments, digital twins address key challenges such as real-time monitoring, 3D visualization, and remote manipulation [10].

The conceptual foundation of digital twin (DT) dates back to 2002, when Dr. Michael Grieves first introduced the “Mirrored Space Model” in a Product Lifecycle Management (PLM) course at the University of Michigan. In 2003, he formalized its architecture as a triad comprising physical entities, virtual models, and data linkages (although the term “digital twin” had not yet been coined) [11,12]. Around 2010, the National Aeronautics and Space Administration (NASA) systematically applied the digital twin concept to the real-time health monitoring of spacecraft in a technical report. A pivotal milestone occurred in 2012, when NASA and the U.S. Air Force Research Laboratory (AFRL) jointly defined the digital twin paradigm in a landmark publication, establishing it as a core technology for high-fidelity vehicle simulation in complex engineering systems. Digital twin technology plays a vital role in optimizing manufacturing through predictive analytics, lifecycle integration, and virtual factory replication, and is positioned by Grieves as a core driver of Industry 4.0 [13]. Although China started relatively late in digital twin technology, it has witnessed remarkable progress in recent years. The country’s 14th Five-Year Plan explicitly emphasizes accelerating the development of industrial internet and promoting intelligent manufacturing transformation, with digital twin technology identified as one of the key enabling technologies for strategic deployment. Recent years have seen extensive research by scholars on digital twin applications in smart manufacturing. Professor Fei Tao’s team at Beihang University proposed the five-dimensional model in 2018, expanding the framework beyond physical and virtual entities to integrate Services, Digital Twin Data, and Connections [14]. This breakthrough enriched DT’s theoretical foundation and significantly enhanced its applicability in smart manufacturing and cyber–physical systems (CPSs). Propelled by these advancements, digital twin technology has rapidly permeated diverse sectors. As a critical enabling platform, it now underpins real-time monitoring, data-driven decision optimization, and remote visualization, with transformative applications in aerospace, smart manufacturing, smart cities, and healthcare [15,16]. Li [17] proposed a cloud-fog-edge (CFE) collaborative computing-based digital twin framework for smart factories (DT-CFE), addressing limitations of traditional digital twin systems in real-time performance, scalability, and data processing capabilities. This multi-layer distributed architecture reduces computational burdens while improving data upload efficiency and system responsiveness. Meanwhile, Chen [18] developed a service-oriented digital twin architecture specifically for additive manufacturing, which overcomes the poor generalizability and high development costs of conventional DT systems through synergistic interactions among service, model, data, and interface layers, enabling the rapid customization of DT solutions. To facilitate the transition toward smart electric vehicle (EV) charging infrastructure, Yu [19] developed an integrated framework comprising green power generation networks, energy storage networks, and charging networks. As the core technology, digital twins empower this infrastructure with intelligent capabilities, including real-time state perception, adaptive adjustment, remote operation/maintenance, and multi-objective coordination.

Digital twin technology is progressively being integrated into manufacturing systems, with a growing number of scholars combining it with robotic technologies to drive intelligent transformation in the sector. Focusing on the evolution and future direction of digital twin (DT) technology in robotics, Mazumder [20] aimed to systematically analyze the current research trends, application scenarios, and technical challenges, and to propose a framework for the development of the next-generation robotic digital twin. Zhang [21] developed a digital twin system for human–robot collaboration (HRC), leveraging cognitive understanding of human intent to guide robotic interactions. This system ensures safe, flexible, and efficient collaboration in shared workspaces. The authors further proposed the ORMR algorithm, which enhances model performance through data-knowledge fusion to achieve accurate human reconstruction under occluded conditions. While this system realizes bidirectional virtual–physical mapping, it faces limitations such as limited interaction modalities and cumbersome fault detection procedures. Li [22] addressed high absolute positioning errors in industrial robot arms by developing a novel PF-CIBAS calibration system. They improved the Beetle Antennae Search (BAS) algorithm using cubic interpolation (CIBAS) to overcome its tendency for local optima and instability, and integrated it with a particle filter (PF) to suppress noise during kinematic parameter identification. This combined approach achieved significantly higher calibration accuracy, reducing the maximum positioning error by 21.43% compared to state-of-the-art methods on an HSR JR680 robot. Xu [23] proposes a digital twin-based industrial cloud robotics (DTICR) framework to enhance control accuracy in robotic manufacturing systems. By integrating high-fidelity digital models with real-time sensory data, the DTICR enables synchronized interaction between digital and physical robots. Robotic control functions are encapsulated as services, simulated in the digital environment, and then mapped to physical robots for execution. A case study demonstrates that the system achieves effective bidirectional synchronization and supports fine-grained control with good flexibility and scalability. Kuts [24] integrated virtual reality technology with factory environments to conduct the modeling and simulation of diverse industrial equipment, developing a synchronized model for real and virtual industrial robots that was experimentally validated in both virtual reality settings and physical shop floors. Kuts [25] further developed a digital twin–VR interface for industrial human–robot interaction and conducted experiments comparing it with traditional teach pendant control. By integrating gaze tracking, heart rate monitoring, and user surveys, the study introduced a multi-metric evaluation framework to assess operator performance, stress, and interface usability. The results highlight the DT-VR interface’s potential as an effective alternative for immersive, user-centered control in Industry 5.0 settings.

In summary, digital twin technology is progressively being integrated into multiple critical domains of manufacturing, particularly in its convergence with robotic technologies. This integration not only provides robot systems with highly synchronized virtual-physical mapping capabilities but also significantly enhances their visualization, predictive analytics, and autonomous decision-making capacities, thereby laying a solid foundation for advancing intelligent manufacturing. While existing research on digital twin systems has achieved notable results and practical applications, several challenges persist, including high communication latency, limited precision, inadequate 3D visualization, and poor user interactivity. In response to these research trends, this study develops a virtual–physical integrated control platform for industrial robots. The platform is designed to unify multiple functionalities such as virtual–physical interaction, intelligent perception, and efficient control, thereby improving the flexibility and reliability of industrial robots in complex operational tasks.

## 2. System Design of Digital Twin-Based Industrial Robot Control Platform

According to widely accepted definitions, a true digital twin is more than a static digital model; it requires real-time bidirectional data exchange with its physical counterpart. The virtual–real fusion control platform utilizes TCP/IP-based socket communication (Port 4003) to continuously acquire physical robot data at 125 Hz (Section 2.3), including joint angles, velocities, voltages, and alarm logs. This high-frequency data stream ensures that the virtual model in Unity3D dynamically mirrors the physical robot’s state, enabling live monitoring and deviation compensation. Beyond passive monitoring, the system implements bidirectional control. Motion commands (e.g., target coordinates or joint angles) generated via the Unity3D interface are packaged and transmitted via the same TCP/IP socket to the physical robot’s control cabinet (Section 2.4). This drives the actual robot’s movement, closing the control loop and enabling remote operation. Bidirectional mapping forms the foundation for considering the system a true digital twin rather than a traditional simulation.

**Figure 1:**
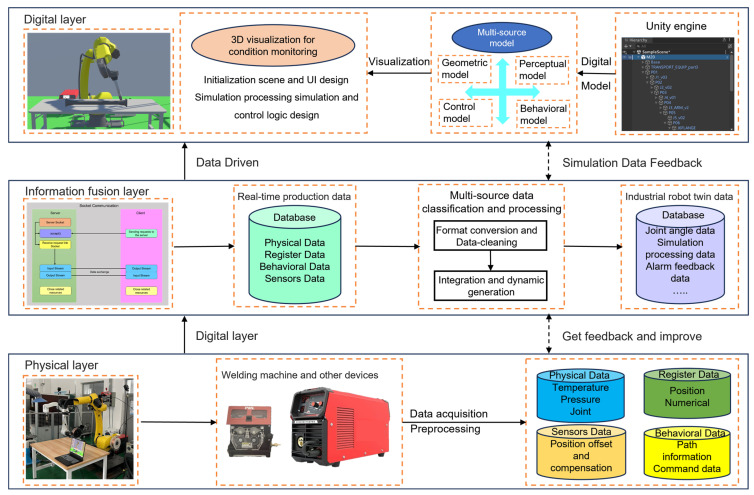
Architecture of the virtual–physical mapping system based on digital twin integration for industrial robot control.

According to the digital twin five-dimensional model to build the industrial robot control platform from three levels, its dimensional architecture is shown in Figure 1, comprising the following:

Physical Layer: The physical layer is the base layer of the digital twin system and includes the robot entities, wire feeders, sensors, and other physical devices and production environments.

Digital Layer: The digital layer is a virtual mapping of the physical layer, a 3D simulation environment of the robot’s working process constructed based on Unity3D, which reproduces the physical state of the real space through the combination of virtual models and multi-source data.

Information Fusion Layer: The information fusion layer serves as an intermediary, integrating and transmitting data between the physical layer and the digital layer. It employs the TCP/IP protocol to ensure reliable data transmission and uses socket communication for data exchange. This layer enables the real-time acquisition of physical data from the robot while simultaneously transmitting control commands from the control platform to the robot control cabinet, achieving bidirectional real-time mapping between the two. Human–computer interaction is facilitated through an interface created using Unity3D’s UGUI (Unity Graphic User Interface) system, thereby enabling interaction between the virtual world and the user.

### 2.1. Physical Entity

**Figure 2:**
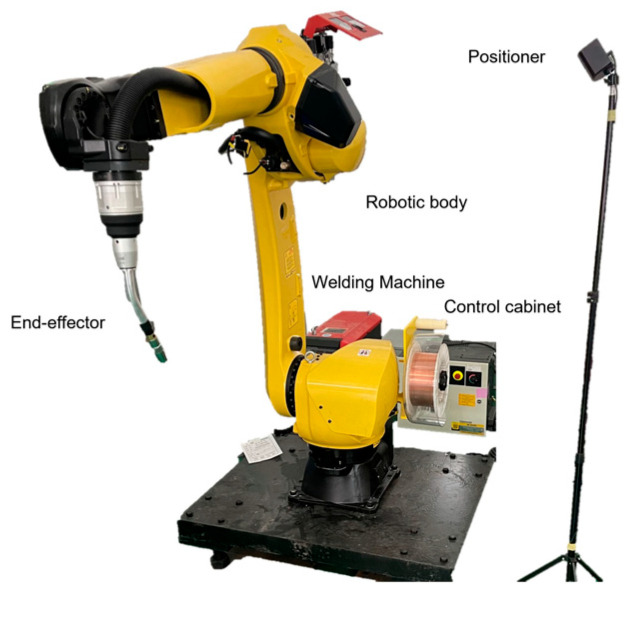
FANUC M-20iD/35 robot.

The physical entity serves as the foundation and key component for constructing the virtual system. It includes the spatial configuration of the robot and its auxiliary equipment. In this study, the FANUC M-20iD/35 industrial robot is selected. This robot features high payload capacity and precision, and it supports the integration of additional equipment such as positioners and force sensors, making it suitable for flexible tasks like precision assembly and grinding. With a repeatability of ±0.08 mm, it meets the requirements of precision manufacturing. The physical entity is shown in Figure 2.

### 2.2. Virtual Model

**Figure 3:**
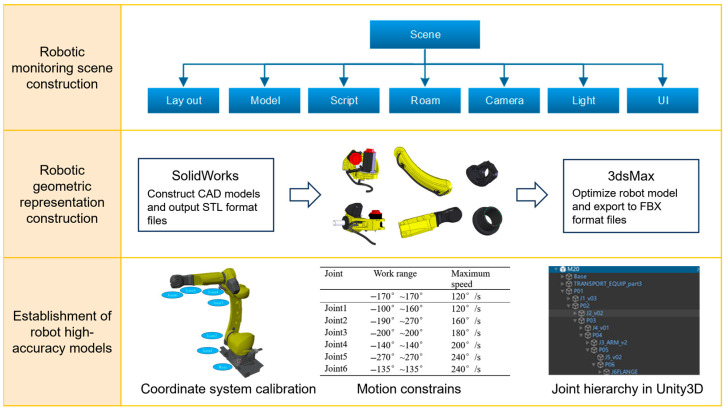
Workflow of digital twin environment construction, including 3D model import, robotic geometry representation, and Unity-based scene layout.

The digital twin environment requires a high-precision 3D robot model to accurately reflect the structure of the physical robot and enable bidirectional mapping between the virtual and physical systems [26]. In this study, the basic virtual robot model was constructed using SolidWorks (2021). Model details and materials were then optimized in 3ds Max, where the coordinate system was converted and the mesh entity generated. The model was subsequently imported into Blender for further adjustments, including the establishment of parent–child relationships among components, allowing child objects to follow the transformations (translation, rotation, and scaling) of their parent objects. Each joint’s rotation center was calibrated to support posture solving (forward and inverse kinematics), enabling the precise trajectory control of the virtual robot’s end effector. Finally, the model was exported in FBX format and imported into Unity3D, resulting in a high-precision digital twin robot model. The construction of the digital twin environment is shown in Figure 3.

### 2.3. Twin Data Acquisition and Input

Bidirectional data communication between the client and server is achieved using the TCP/IP protocol and socket communication, establishing a connection between the physical robot and the digital twin environment to enable two-way physical–virtual mapping. The communication process is illustrated in Figure 3. To ensure network compatibility, both the robot controller and the host PC were assigned IP addresses within the same subnet. The FRRJIF.DLL library was imported into the Unity environment, whereby custom scripts were written to instantiate the FRRJIF.Core object. Subsequently, a DataTable object was configured to incorporate required data types, including status information of various components such as joint angles, Cartesian coordinates, system variables (registers and status flags), velocities, voltages, currents, and alarm logs. The DataTable.Refresh() method was iteratively invoked within a loop to continuously poll robot state data at a refresh rate of 125 Hz. The twin system continuously monitors the robot’s pose information to determine whether the positional deviation falls within the threshold range. If within tolerance, the system sequentially issues command feedback to the robot controller; if exceeding limits, it first corrects the pose deviation. By continuously monitoring twin data, the system periodically evaluates the robot’s condition and identifies potential faults in advance [27,28,29,30,31].

The script writes data to a designated port, where the socket packages the command data into a transmission-friendly format (such as JSON or binary). This data is then transmitted via the TCP/IP protocol to the control cabinet of the physical robot, guiding it to perform the specified actions. In this way, the digital twin platform gains control over the physical robot. As the physical robot executes these actions, its control cabinet sends updated status information back to the digital twin platform, realizing bidirectional mapping between the virtual and physical systems. This virtual–physical interaction enables the digital twin robot to achieve precise control, real-time monitoring, and remote maintenance, thereby enhancing the system’s level of intelligence.

### 2.4. Design of the Human–Machine Interaction Interface

Unity3D is a widely used, powerful, flexible, and cross-platform game development engine. Its built-in physics engine supports various physical features such as rigid bodies, collision detection, and joints, making the digital twin world more realistic. Originating from game development, Unity3D offers strong rendering capabilities and high extensibility, making it an ideal choice for industrial digital twin platforms [32].

**Figure 4:**
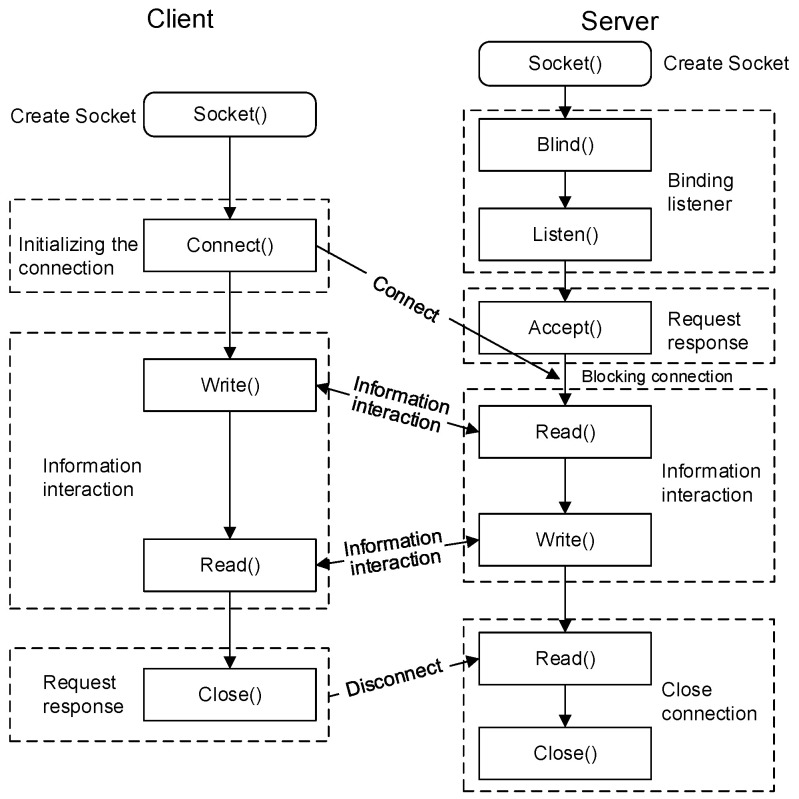
Socket-based client–server communication process between the digital twin platform and the robot controller.

In Unity, C# is the primary programming language used for writing logic, interaction functions, and system scripts. In this study, C# and Unity3D are used to develop the functional interface of the robot digital twin system. The system listens to and parses TCP data in real time. At the same time, the twin platform sends motion commands (such as coordinates or joint angles) based on user input to drive the movement of the virtual robot. The human–machine interaction interface is shown in Figure 4.

**Figure 5:**
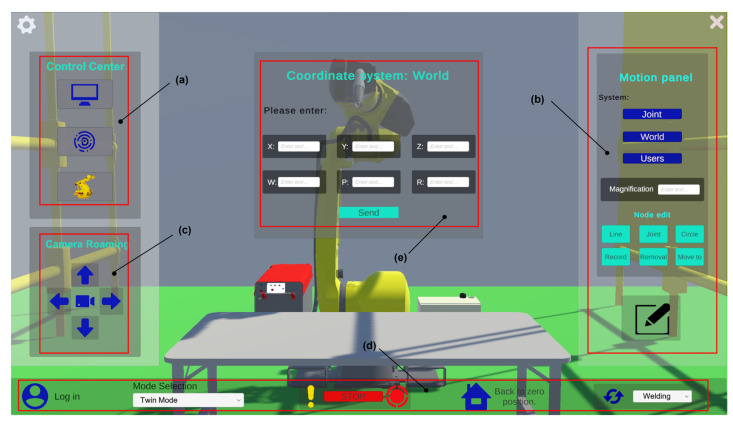
Graphical user interface design of the digital twin platform, illustrating the layout and core functionalities of human–robot interaction modules.

As shown in Figure 5:

Panel (a) contains buttons for opening the Control Center, Communication, and Device Management.

Panel (b) is the motion panel, which integrates core functions for robot motion control and program development. Through this panel, users can write robot programs and simulate them in the digital twin platform. Motion commands are transmitted via the TCP/IP protocol and synchronized with the physical robot’s control cabinet. Users can control the robot’s movements using three coordinate systems: joint, world, and custom. For example, clicking the World Coordinate System button opens interface (e), where users can control the robot’s pose in the world coordinate system. The system calculates the corresponding joint angles through inverse kinematics and drives the robot to the target pose.

Panel (c) is the view navigation panel. The mouse scroll wheel zooms in/out, the arrow keys pan the view, and the right mouse button rotates the camera. Pressing the “R” key resets the current camera position. Clicking the center button opens the visual sensor window, enabling better monitoring of the machining process.

Panel (d) is the integrated panel, which includes key functions such as login, mode switching, emergency stop, home position, and end-effector configuration. Login: Verifies user permissions and identity; Mode switching: Allows switching between simulation mode, twin mode, and other operational modes; Emergency stop: Immediately halts the robot in case of emergencies to ensure safety; Home position return: Instantly returns the robot to a preset initial pose for task re-alignment; End-effector configuration: Supports parameter setup and switching of end tools (e.g., grippers and welding torches).

This panel (e) provides users with the main access point and critical control features for the digital twin platform and plays an essential role in ensuring stable system operation. With the completion of the above steps and by leveraging Unity3D’s powerful physics engine and rendering capabilities, the system not only offers excellent human–machine interaction and visual performance but also supports the real-time monitoring and adjustment of the simulation process.

## 3. Development and Integration of Motion Simulation for Digital Twin Industrial Robot Systems

### 3.1. Research on Robot Path Planning Algorithms

Trajectory planning for digital twin robots is based on forward and inverse kinematics analysis. It generates a reasonable sequence of motions according to specific task requirements, guiding the end-effector to transition smoothly from the initial pose to the target pose while satisfying obstacle avoidance and other motion constraints. Trajectory planning strategies are mainly divided into two categories, joint space-based planning and Cartesian space-based planning, each suited to different control requirements and application scenarios.

#### 3.1.1. Joint Space-Based Trajectory Planning

Trajectory planning in joint space requires first obtaining the pose matrices of the initial point, intermediate points, and target point that the end-effector passes through. Then, inverse kinematics is used to calculate the corresponding joint angles. Finally, interpolation methods such as polynomial interpolation are employed to connect the initial, intermediate, and target joint angles into a smooth function curve.

Taking cubic polynomial interpolation as an example, the joint angle $\dot{\theta }$ is expressed as a function of time t, as shown in Equation (3), where *θ*_0_ represents the joint angle at the initial time *t*_0_, and *θₑ* represents the joint angle at the final time tₑ.

(1) $$\theta \left(t\right)=a_{0}+a_{1}t+a_{2}t^{2}+a_{3}t^{3}$$

Using the four constraints on the joint angles and angular velocities at the initial and termination points, it is possible to find:

(2) $$\left\{\begin{array}{c} a_{0}=\theta_{0}\\ a_{1}={\dot{\theta }}_{0}\\ a_{2}=\frac{3\left(\theta_{e}-\theta_{0}\right)}{t_{e}^{2}}-\frac{2{\dot{\theta }}_{0}+{\dot{\theta }}_{e}}{t_{e}}\\ a_{3}=\frac{-2\left(\theta_{e}-\theta_{0}\right)}{t_{e}^{3}}-\frac{{\dot{\theta }}_{0}+{\dot{\theta }}_{e}}{t_{e}^{2}} \end{array}\right.$$

The fifth-degree polynomial interpolation is similar to the third-degree polynomial interpolation, but it needs to satisfy six constraints, such as the joint angles, angular velocities, and angular accelerations at the initial and termination points.

#### 3.1.2. Trajectory Planning Based on Cartesian Space

**Figure 6:**
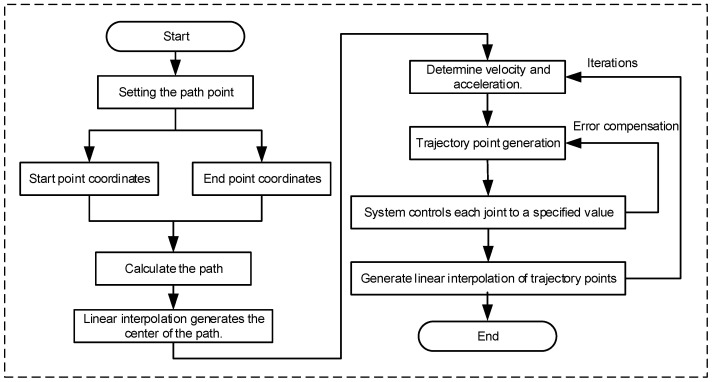
Interpolation compensation trajectory planning flowchart.

When performing trajectory planning in joint space, the system adjusts the joint angles to eventually guide the end-effector to the desired pose. However, since this method does not directly constrain the end-effector’s pose in the workspace, it may be less precise in controlling its motion path. In contrast, Cartesian space trajectory planning takes the end-effector’s position and orientation in the workspace as the direct planning targets, allowing for the precise control of its movement path. Therefore, it is more suitable for applications with strict requirements on trajectory shape, see Figure 6.

After determining the poses of the end-effector’s initial position P_S_(x_s_, y_s_, z_s_) and target position P_e_(x_e_, y_e_, z_e_), the trajectory between the two points is discretized into a path composed of multiple interpolation points. By calculating the pose coordinates of these interpolation points, the end-effector can pass through each point in sequence, thereby achieving an approximately continuous linear motion. This approach is commonly used in tasks that require high precision in the end-effector’s pose, such as welding, grinding, and material handling.

The length *L* of the linear trajectory from Ps to Pe is:

(3) $$L=\sqrt{{\left(x_{e}-x_{0}\right)}^{2}+{\left(y_{e}-y_{0}\right)}^{2}+{\left(z_{e}-z_{0}\right)}^{2}}$$

The coordinates of the interpolation points are:

(4) $$\left\{\begin{array}{c} {\;x}_{i}={\;x}_{0}-\frac{x_{e}-x_{0}}{N}i\\ {\;y}_{i}={\;y}_{0}-\frac{y_{e}-y_{0}}{N}i\\ {\;z}_{i}={\;z}_{0}-\frac{z_{e}-z_{0}}{N}i \end{array}\right.$$

where *i* is 1, 2,…, N denotes the interpolation point, and N is the total number of interpolation points.

### 3.2. Design of a Robot Control Center with a Real–Virtual Synchronization Mechanism

To achieve high-precision and low-latency synchronization between the physical robot and the digital twin system, this study integrates an out-of-step detection and consistency verification mechanism within a centralized control panel. A predefined threshold is set to identify deviations between the virtual and physical systems. During each sampling cycle, the physical robot transmits real-time data—including joint angles, tool coordinates, and alarm logs—to the control center. This ensures that the digital twin remains closely aligned with the actual robot’s state, while also enabling robust fault-tolerant handling in cases of network delays or data loss.

**Figure 7:**
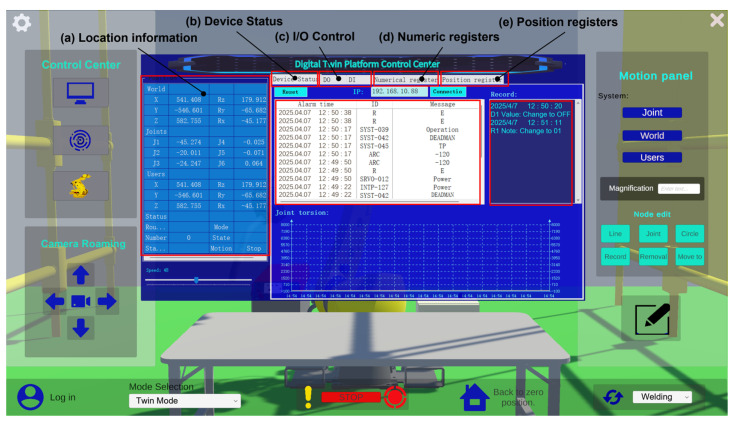
Digital twin platform control center.

The control center integrates several key functional modules, as shown in Figure 7, whose functions mainly cover robot position information, device state management, I/O signal control, register data operation, etc., which provides strong technical support for the whole digital twin system.

The control center collects and manages the robot’s position and joint angle data in real time, transmitting it to the position interface as shown in Figure 7a. This data is also synchronized with the digital twin model to enable real-time status updates. In the device status interface, users can access the Alarm Buffer in Figure 7b, which provides detailed information such as alarm ID, severity level, alarm description, and trigger time. This information is visualized in the Unity front-end interface to assist users in rapid diagnosis and maintenance. The I/O signal control interface, as illustrated in Figure 7c, allows for the real-time reading of the I/O interface status and supports the management and control of the robot’s I/O signals. To enhance readability and maintainability, the system allows users to add annotations to each I/O signal, enabling labeling and function prompts directly within the interface. The register management interfaces shown in Figure 7d,e support the interaction with and management of the robot’s internal numeric registers (R\[]) and position registers (PR\[]). Users can assign names and usage descriptions to each register point in the interface, facilitating operations such as trajectory presetting and position recognition.

Through the coordinated operation of the above modules, the system achieves real-time integrated control from low-level control to high-level visualization, laying a solid foundation for the application of digital twin technology in industrial-grade robotics.

### 3.3. Packaging and Motion Testing of Functions

Building upon the previously described trajectory planning algorithm, it is further encapsulated into a MATLAB (R2021a) function and compiled into a corresponding DLL library interface for seamless invocation within the C# environment and integration into the digital twin control platform. A virtual workstation is established using Roboguide, where the industrial robot control platform connects to the virtual robot via a loopback address. This setup enables motion testing without relying on physical equipment, effectively reducing debugging costs and preventing potential damage to actual hardware during trial-and-error processes.

**Figure 8:**
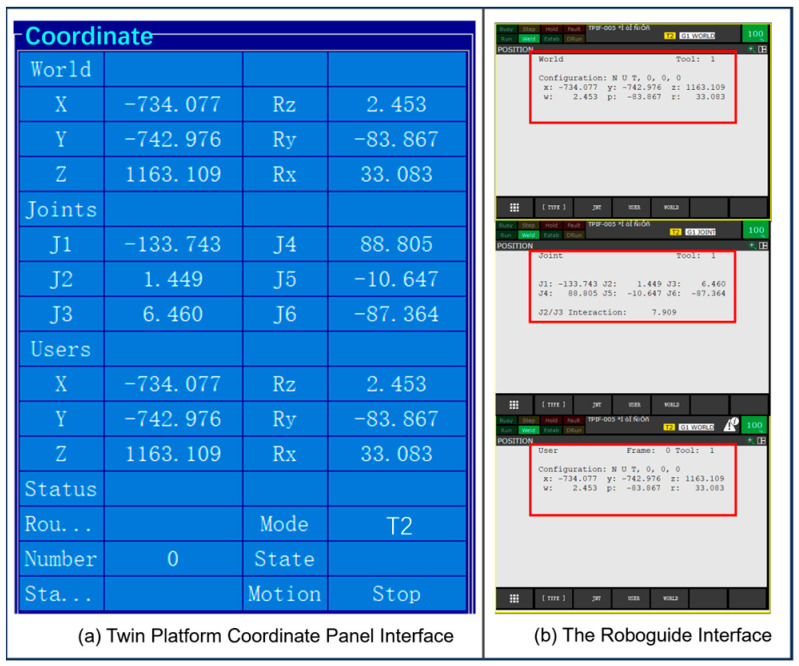
Position information at a certain moment of the motion process during the test.

After initial motion testing, the system achieves the real-time synchronization of the robot’s movements. Figure 8a and Figure 8b show the position information of the digital twin robot in the control platform and the joint and world position data in the virtual teach pendant, respectively.

## 4. Space Free-Form Surface Welding Experiment

According to statistics from the International Federation of Robotics (IFR), welding is the second-largest application of industrial robots, following material handling [33,34,35]. How to intelligently control robots to perform welding tasks efficiently and with high quality has long been a key research focus in the welding field. To verify the system’s motion accuracy and real-time mapping performance on complex surfaces, a welding experiment was designed and conducted on a three-blade helical propeller.

**Figure 9:**
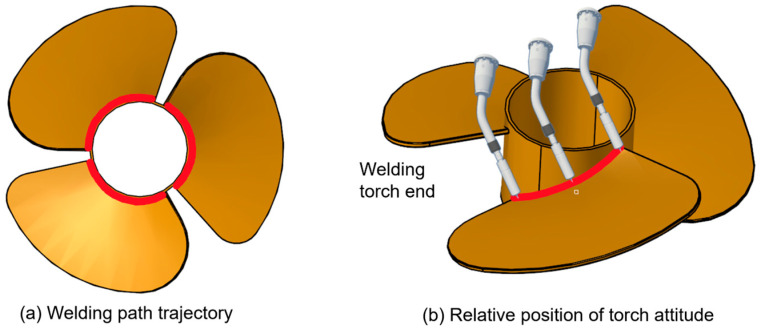
Relative position of the torch end to the part.

A schematic diagram of the welding path for this part is shown in Figure 9 to help readers better understand the robot’s welding trajectory. It clearly illustrates the relative position between the welding torch tip and the workpiece. The red curve represents the TCP path in three-dimensional space, providing an intuitive visualization of the robot’s motion posture during operation. 

**Figure 10:**
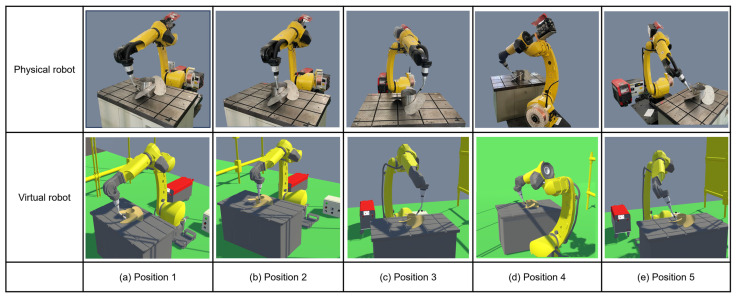
Different positions of the robot’s curved motion.

Through online welding trajectory programming via the control platform, the system enables the real-time monitoring of status information and the robot’s end-effector pose during the welding process. The robot’s positions along different points of the curved trajectory are shown in Figure 10.

Table 1 records the coordinates of the five positions shown in Figure 10 as well as other trajectory points and information about the robot joints (J1~J6) (Table 1 records nine positions of the robot moving along the welding trajectory, and these positions contain the five positions shown in Figure 10). The coordinate values of the physical robot in the X, Y, and Z directions and the angle values of the six joints were recorded by reading the data on the Fanuc robot demonstrator, and the twin robot spatial position information and joint angles can be read in the position panel.

**Figure 11:**
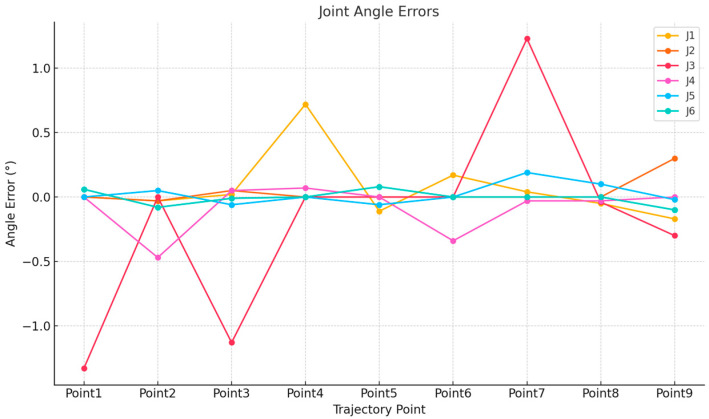
Comparative analysis of joint angle deviations: error profiles across six robotic joints (J1–J6) between simulated and physical systems.

**Figure 12:**
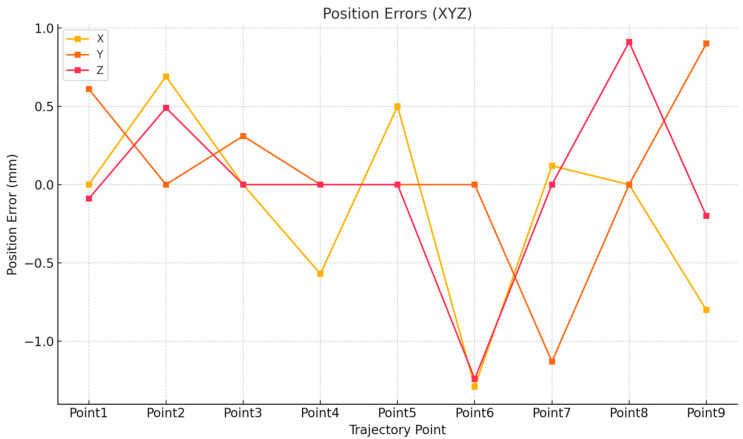
Comparative analysis of positional trajectory deviations: three-dimensional axial error profiles (X, Y, and Z) for simulated vs. physical robotic systems.

Table 1 presents the spatial position information of the robot’s motion trajectory points along with the corresponding joint angle data. The comparative analysis between the virtual and physical robotic systems reveals high overall consistency, with minor deviations observed in both joint angles and end-effector positions (Figure 11 and Figure 12). The joint angle errors across J1–J6 remain within acceptable tolerance ranges, indicating a high level of control accuracy. For spatial positioning, the X, Y, and Z axis errors are mostly within ±1 mm, confirming the effectiveness of the virtual–physical synchronization. These deviations may result from minor vibrations during robot operation and differences in data acquisition methods. It is also worth noting that the experiment was conducted in a controlled laboratory environment with minimal external disturbances, which helped ensure stable communication and accurate data exchange. Overall, such minor differences are considered acceptable within the context of a digital twin robot system.

## 5. Conclusions

This study aims to lower the operational threshold of traditional industrial robots and enhance the standardization of their control processes. A virtual–physical mapping architecture was constructed, encompassing the physical layer, digital layer, and information fusion layer. By innovatively introducing digital twin technology, a high-precision industrial robot control platform was designed and built. Leveraging a graphical interface and modular commands, the platform effectively simplifies the programming process of industrial robots. To verify the reliability of the platform, the welding of spatial surface components was used as the application scenario for experimental validation. The results demonstrated a high degree of consistency between the virtual and physical data, validating the platform’s reliability in supporting curved surface operations.

The proposed cyber–physical control platform significantly improves the intelligence of industrial robots in complex tasks and offers strong scalability and versatility. It enhances processing efficiency while reducing the risk of safety incidents. Although there is still room for improvement, future work may focus on areas such as stress–strain analysis, multi-robot collaboration, and integration with cloud-based big data. These directions will provide solid technical support for the advancement of intelligent robotics and enable higher-level industrial intelligence applications.

## Figures and Tables

**Table 1:** Fanuc robot motion trajectory point positions and joint angles.

Position/Joint Angle	Physical/ Virtual	Trajectory Point 1	Trajectory Point 2	Trajectory Point 3	Trajectory Point 4	Trajectory Point 5	Trajectory Point 6	Trajectory Point 7	Trajectory Point 8	Trajectory Point 9
X/mm	Physical	415.40	351.40	408.34	243.51	295.74	390.15	388.15	−641.44	390.65
	Virtual	415.40	350.71	408.34	244.08	295.24	391.44	388.03	−641.44	391.45
Y/mm	Physical	546.42	402.98	469.51	431.79	603.78	408.51	411.38	380.21	536.12
	Virtual	545.81	402.98	469.20	431.79	603.78	408.51	412.51	380.21	535.22
Z/mm	Physical	−50.30	−156.58	−114.14	−113.37	−88.72	−108.41	−104.34	−124.28	−92.33
	Virtual	−50.21	−157.07	−114.14	−113.37	−88.72	−107.17	−104.34	−125.19	−92.13
J_1_/(°)	Physical	2.09	13.02	8.95	28.57	−17.61	14.35	7.65	12.42	19.21
	Virtual	2.09	13.05	8.93	27.85	−17.50	14.18	7.61	12.47	19.38
J_2_/(°)	Physical	21.08	12.41	17.08	6.19	−6.54	31.15	16.04	18.43	25.43
	Virtual	21.08	12.44	17.03	6.19	−6.54	31.15	16.04	18.43	25.13
J_3_/(°)	Physical	−23.84	−18.29	−23.84	−29.47	25.34	−24.17	−15.19	−27.15	−23.59
	Virtual	−22.51	−18.29	−22.71	−29.47	25.34	−24.17	−16.42	−27.11	−23.29
J_4_/(°)	Physical	−3.80	−11.27	−7.63	34.89	−42.61	−11.69	−15.83	−38.42	−13.21
	Virtual	−3.80	−10.80	−7.68	34.82	−42.61	−11.35	−15.80	−38.39	−13.21
J_5_/(°)	Physical	−75.46	−74.37	−73.66	−71.84	−48.47	−64.32	−76.93	−63.72	−73.12
	Virtual	−75.46	−74.42	−73.60	−71.84	−48.41	−64.32	−77.12	−63.82	−73.10
J_6_/(°)	Physical	9.97	26.20	28.41	3.06	15.19	5.69	21.43	32.16	31.32
	Virtual	9.91	26.28	28.42	3.06	15.11	5.69	21.43	32.16	31.42

## Data Availability

The data that support the findings of this study are available from the corresponding author upon reasonable request.
